# Rare giant female adnexal tumor of probable Wolffian origin: a case report

**DOI:** 10.1093/jscr/rjac243

**Published:** 2022-05-27

**Authors:** Sanela Brzika, Ismar Rašić, Admir Bektešević, Ali Gavrankapetanović, Nedim Hasić, Salko Pašović

**Affiliations:** Department of Surgery, General Hospital, Sarajevo 71 000, Bosnia and Herzegovina; Department of Surgery, General Hospital, Sarajevo 71 000, Bosnia and Herzegovina; Department of Surgery, General Hospital, Sarajevo 71 000, Bosnia and Herzegovina; Department of Surgery, General Hospital, Sarajevo 71 000, Bosnia and Herzegovina; Department of Surgery, General Hospital, Sarajevo 71 000, Bosnia and Herzegovina; Department of Surgery, General Hospital, Sarajevo 71 000, Bosnia and Herzegovina

## Abstract

Female adnexal tumors of probable Wolffian origin (FATWOs) are extremely rare tumors, with only around 100 cases published worldwide. FATWOs are most frequently found in the broad ligament, but these can also appear in the mesosalpinx, Fallopian tube, ovary, paravaginal region or peritoneum. We present a case of a 68-year-old female with a history of painless abdominal distension and frequent urination, with palpable big abdominal mass. Initial diagnosis was made using ultrasound and computed tomography scan that showed 22 × 21-cm tumor with solid and cystic components. Blood test showed elevated serum levels of CA 125, HE4 and Roma index. Intraoperatively, a large abdominopelvic encapsulated mass, fixated to surrounding tissue, was found. Our patient underwent hysterectomy 12 years ago. Total tumor resection, including bilateral adnexectomy, was performed. At 6 months of following, there was no evidence of disease. Here, we report extremely rare abdominal tumors and one of the biggest FATWOs reported so far.

## INTRODUCTION

Kariminejad and Scully [1] first reported the occurrence of female adnexal tumors of probable Wolffian origin (FATWOs) in 1973, and they regarded it as a non-malignant lesion. This rare type of neoplasm typically arises from the persisting remnants of the mesonephric duct following its natural degeneration. These epithelial tumors are extremely rare, with only around 100 cases published worldwide [2]. FATWOs are most frequently found in the broad ligament, but these can also appear in the mesosalpinx, Fallopian tube, ovary, paravaginal region or peritoneum [3]. The median age at diagnosis is 50 years (range: 15–87 years) [2]. Although it is generally considered as a benign entity, recurrent and metastatic cases have been reported. Large tumor size, capsular invasion with rupture, increased mitotic activity, hypercellularity and nuclear atypia indicate aggressive clinical course [3]. Generally, the most adequate treatment is considered to be total surgical resection, total hysterectomy and bilateral salpingo-oophorectomy. Due to its malignant potential, regular and long-term follow-up is recommended [4].

## CASE REPORT

**Figure 1:**
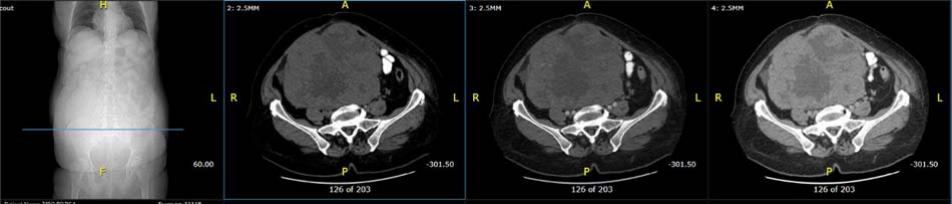
Axial computed tomography (CT) scan shows solid mass in abdominopelvic cavity.

**Figure 2:**
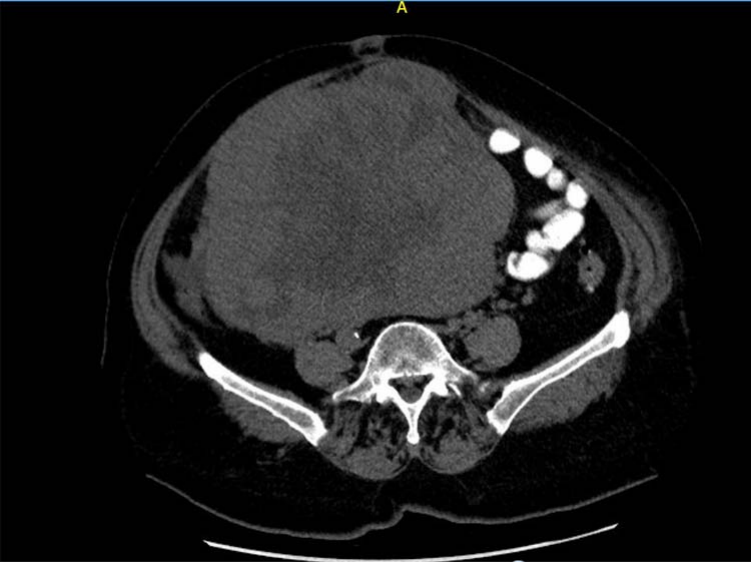
Close picture of axial CT scan of tumor, showing more right-sided localization.

**Figure 3:**
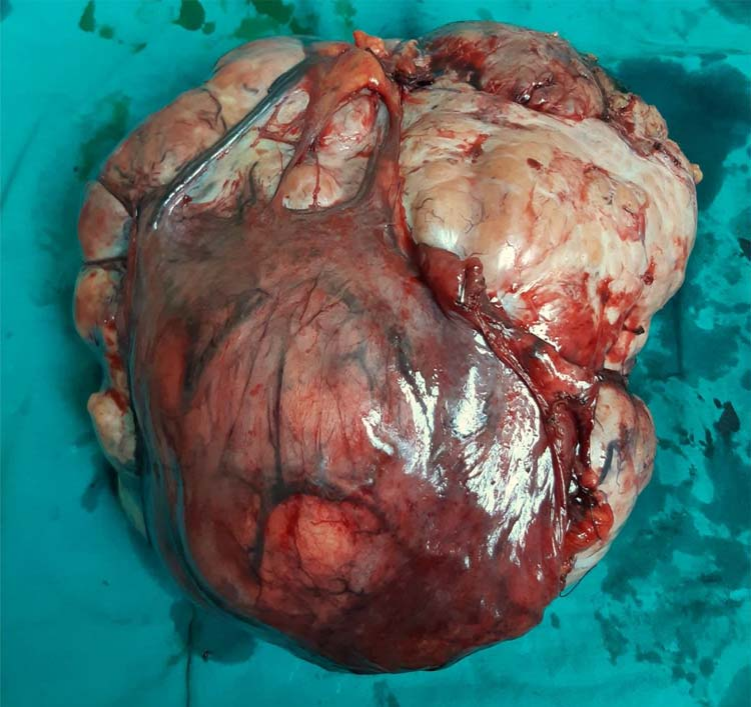
Surgically removed tumor mass that was sent to pathology, mostly right ovary and adnexa with unremarkable cervix.

**Figure 4:**
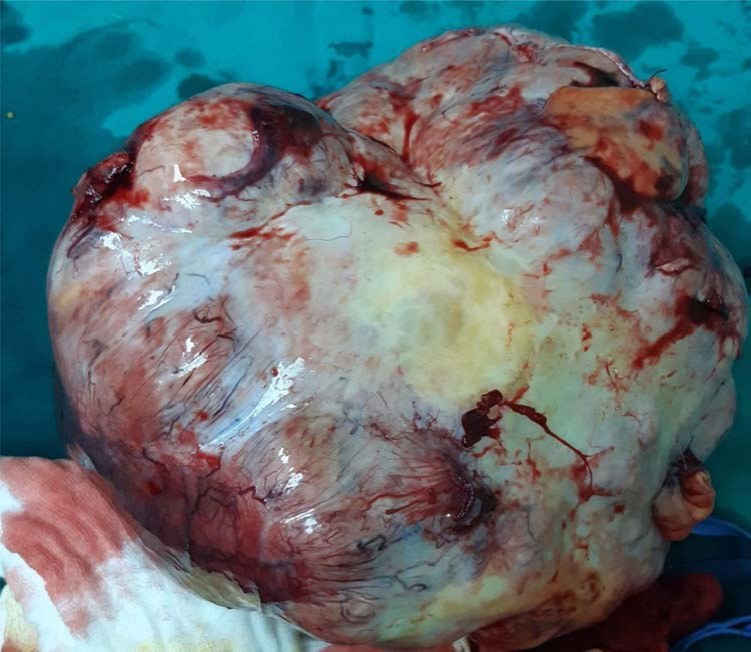
Surgically removed tumor mass, anterior side view.

**Figure 5:**
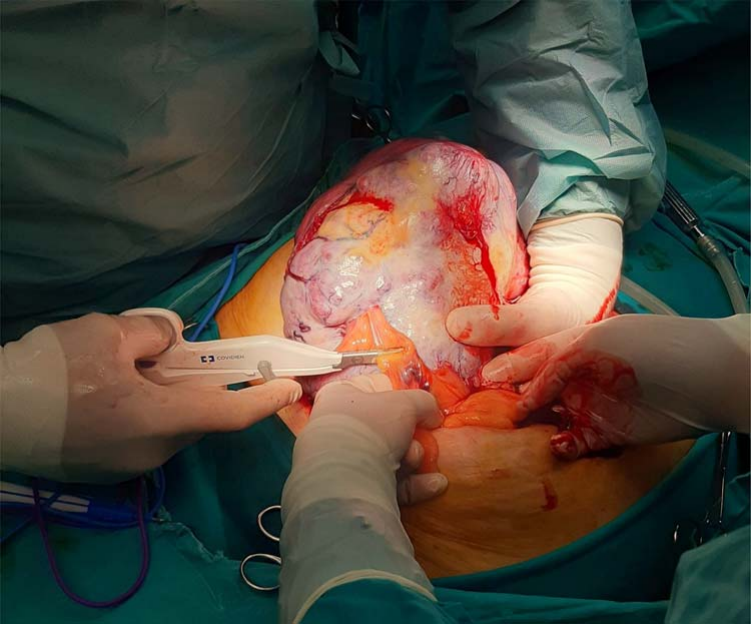
Intraoperative image of removal of the tumor from surrounding tissue and neovascularization: large mass identified following opening of the peritoneal cavity.

A 68-year-old female patient presented with a palpable abdominal mass in the lower parties of the abdomen. Three months earlier, she noticed painless abdominal distension and frequent urination. She underwent hysterectomy without oophorectomy for treatment of uterine myoma 12 years ago. The results of the transabdominal ultrasound and cross-sectional scan showed a 22 × 21-cm abdominopelvical mass, more right-sided, with both solid and cystic components, and incipient hepatomegaly with single hypodense lesion of liver, 8 mm in diameter. Blood test showed elevated serum cancer antigen 125 (CA 125) 185.9 U/ml, human epididymis secretory protein 4 (HE4) 228.3 pmol/l levels, and the risk of ovarian malignancy algorithm index (Roma index) was high with 79.9%. After a laparotomy, large tumorous mass 22 × 21 cm in diameter was found, which was grayish brown to yellowish color with nodular surface and cystic components, occupying the entire abdominopelvic cavity. (Figs 1 and 2) The tumor was well encapsulated and solid, fixated to the omentum and mesentery with large, nutritional, blood vessels and to surrounding tissue (Figs 3–5). Complete surgical resection of tumor, including bilateral adnexectomy, was performed. The post-operative period was uneventful. After 6 months of follow-up, magnetic resonance imaging of the abdomen and pelvis was performed, and there were no signs of tumor recurrence and serum levels of tumor markers were in reference ranges.

Microscopic analysis showed tubular, insular and cribriform tumor cells which were separated with bands of fibrous, well-vascularized, partially hyalinized stroma with cells of predominantly uniform round to oval nuclei. The cytoplasm of tumor cells was eosinophilic in places and transparent with noticeable vacuoles. Nuclear atypia was mild to moderate. Mitotic activity was low (up to one-tenth to three-tenth high-powered fields). Tumor tissue showed cystic degenerative changes and necrosis, and tumor cells showed positive marker on cytokeratin 19 (CK19), cytokeratin AE1/AE3, cytokeratin 18 (CK18), rare single cells on CK5/6 and CK7, inhibin, vimentin and epithelial membrane antigen (EMA). Histological examination of the liver lesion revealed a typical cavernous hemangioma.

## DISCUSSION

Female adnexal tumor of probable Wolffian origin has been described to have a varied morphology, which was highlighted in the series reported by Kariminejad and Scully [1]. Less than 100 cases of FATWO have been reported, and the majority show benign behavior [2].

 In the 23 described FATWO by Sinha, Risha *et al*. [5] the median tumor size in greatest dimension was 12.16 cm, which positioned tumor described in our case report in the group with largest dimensions, and operative findings were notable for right laterality being more prevalent. According to the Ramirez *et al*.’s [6] review, it has been found that, preoperatively, serum CA 125 levels were normal in all of the patients. In our case, blood test showed elevated serum CA 125, HE4 levels and Roma index. Although routine tumor markers for ovarian malignancy do not have much role in the diagnosis of FATWO, and in most of described cases, preoperative values of serum tumor markers were within the reference values, in some reported cases of recurrent disease, the serum levels of CA 125 and CA 19–9 were elevated [7, 8].

The described macroscopic characteristics based on literature review correspond to our case. FATWOs are sharply demarcated and encapsulated tumors. They are often solid gray-yellow or brownish tumors with areas of hemorrhage and cystic necrosis [3]. There was similarity with our case, where the nuclear atypia was mild to moderate and mitotic activity was low, which is important because increased mitotic activity, hypercellularity and nuclear atypia indicate aggressive clinical course, as was mentioned before.

Devouassoux-Shisheboran *et al*. [9] found in a larger study of 25 cases that FATWOs are immunoreactive for pancytokeratin AE1/3 (100%), cytokeratin 5.2 (100%), vimentin (100%), calretinin (91%), cytokeratin 7 (88%), α-inhibin (68%) and EMA (12%). The immunotype of AE1/3, vimentin, EMA, calretinin and inhibin of our case was consistent with those described in most literature reports. Tumor cells showed positive marker also on CK19, CK18 and rare single cells on CK5/6 and CK7.

The recurrence could occur in the absence of the aggressive histological character and even after several years following the initial diagnosis [10]. The prognosis of this tumor does not correlate with their clinical presentation and their cytology [11]. Recurrence median time was 48 months with a range from 13 to 96 months, and in some cases, recurrences occurred even after a long interval following the diagnosis [6]. Metastasis or recurrences have been reported to occur in ~11% of cases and they may occur as early as 2 years. The most frequent metastatic sites are the liver and the lung [1, 3]. The lesion from the liver that was sent to pathology came negative to tumor cells, and our patient did not have any other signs of metastatic disease.

Standard treatment for Wolffian adnexal tumors is considered to be complete surgical resection, including hysterectomy with bilateral adnexectomy. Adjuvant chemotherapy or radiation therapy was controversial and was typically not an effective treatment. Although fertility-sparing surgery is possible since most tumors are limited to one ovary at presentation, most recurrences have been reported in patients formerly treated by tumor resection alone [11, 12]. After 6 months of following, there were no evidence of the disease.
